# Changes and response mechanism of sugar and organic acids in fruits under water deficit stress

**DOI:** 10.7717/peerj.13691

**Published:** 2022-08-24

**Authors:** Wei-Feng Ma, Yan-Biao Li, Guo-Jie Nai, Guo-Ping Liang, Zong-Huan Ma, Bai-Hong Chen, Juan Mao

**Affiliations:** College of Horticulture, Gansu Agricultural University, Lanzhou, Gansu, China

**Keywords:** Water stress, Sugar and acid metabolism, Enzyme activity, Gene expression, Signal transduction

## Abstract

The content and the ratio of soluble sugars and organic acids in fruits are significant indicators for fruit quality. They are affected by multiple environmental factors, in which water-deficient is the most concern. Previous studies found that the content of soluble sugars and organic acids in fruit displayed great differences under varied water stress. It is important to clarify the mechanism of such difference and to provide researchers with systematic knowledge about the response to drought stress and the mechanism of sugar and acid changes in fruits, so that they can better carry out the study of fruit quality under drought stress. Therefore, the researchers studied dozens of research articles about the content of soluble sugar and organic acid, the activity of related metabolic enzymes, and the expression of related metabolic genes in fruits under water stress, and the stress response of plants to water stress. We found that after plants perceived and transmitted the signal of water deficit, the expression of genes related to the metabolism of soluble sugars and organic acids changed. It was then affected the synthesis of metabolic enzymes and changed their metabolic rate, ultimately leading to changes in soluble sugar and organic acid content. Based on the literature review, we described the pathway diagrams of sugar metabolism, organic acid metabolism, mainly malic acid, tartaric acid, and citric acid metabolism, and of the response to drought stress. From many aspects including plants’ perception of water stress signal, signal conversion and transmission, induced gene expression, the changes in soluble sugar and the enzyme activities of organic acids, as well as the final sugar and acid content in fruits, this thesis summarized previous studies on the influence of water stress on soluble sugars and the metabolism of organic acids in fruits.

## Introduction

Drought is among the most important abiotic limiting factors in agricultural production. Drought stress alone has caused about $30 billion losses in global crop production over the past decade (Gupta, Rico-Medina & Cao-Delgado, 2020). Drought is one cause of water stress, whereby water loss exceeds water absorption, resulting in a decrease in water content and turgor pressure in plant tissues, and abnormal metabolism. In fruit crop production, water stress may cause severe reduction in fruit quality and yield. Therefore, it is important for improvement of fruit quality under water stress to understand the mechanism of metabolic changes.

In fruit, soluble sugars mainly comprise fructose, sucrose, and glucose, of which fructose is the sweetest (Wang et al., 2018, 2019). Organic acids in fruit predominantly comprise malate, citrate, and tartaric acid (Chen et al., 2015). The sugar:acid ratio is the primary index for evaluation of fruit quality, specifically of flavor and fruit ripening (Zhang et al., 2021). In addition, soluble sugars are substrates for anthocyanin synthesis, and acids are a potential stimulus for anthocyanin synthesis, which is important for fruit coloring (Noro, Kudo & Kitsuwa, 1989; Smeekens, 2000; Ban et al., 2009; Lester, Jifon & Makus, 2010). Therefore, understanding the mechanisms of sugar and acid metabolism is important for improvement of fruit quality under water stress.

Isotope labeling has revealed that the O_2_ gas and H^+^ ions produced by photosynthesis are derived from water, and the H^+^ ions are utilized for carbon assimilation. Water acts as a solvent to transport organic compounds synthesized by photosynthesis between source and sink units, and plays an important role in fruit sugar metabolism. In addition, the synthetic substrate of organic acids in plants is the sugar produced from photosynthesis. Therefore, sugar and organic acid metabolism are closely related. Researchers have long studied the effect of water stress on sugar and acid metabolism in fruit (Li, Feng & Cheng, 2012; Jiang et al., 2020). Genetic modification, osmoregulation, and the breeding of drought-resistant cultivars have been used to lessen the impact of water stress on fruit sugar and acid metabolism, and to promote the orderly metabolism of fruit sugars and acids. In addition, this research has provided insight into the mechanisms of sugar and acid metabolism under water stress.

Moderate water deficit has a positive effect on the soluble sugar content and sugar:acid ratio of the fruit of jujube (*Ziziphus jujuba* Mill.) ‘Lizao’ (Cui et al., 2009). However, drought stress has a negative impact on the jujube ‘Lingwuchangzao’ fruit sugar:acid ratio and pigment content (Jiang et al., 2020). Water stress improves the activities of sucrose synthase (SuSy) and invertase (INV) in tomato fruit (Lu, Li & Jiang, 2009). With regard to acid metabolism, organic acids in fruit can be roughly divided into tartaric acid, malate, and citrate types. L-Idonate dehydrogenase (L-IDN DH) catalyzes the cleavage between the C4 and C5 positions of ascorbic acid (AsA), thus resulting in the synthesis of tartrate (Narnoliya, Kaushal & Singh, 2019). Malate, citrate, and other organic acids are synthesized through the tricarboxylic acid (TCA) cycle (Huang et al., 2021). Organic acid accumulation in sarcocarp cells is under environmental control. Water stress has no notable effect on titratable acid content (Reynolds & Naylor, 1994). Ussahatanonta, Jackson & Rowe (1996) observed that water deficit leads to an increase in pH and reduction in tartaric acid content in grape (*Vitis vinifera* L.) ‘Cabernet Sauvignon’ berries, whereas drought increases the total acid content in fruit of orange (*Citrus sinensis* (L.) Osbeck) ‘Valencia’ (Hutton, Landsberg & Sutton, 2007). Drought strongly prevents the degradation of citrate in fruit, thereby increasing the citrate content in fruit, in association with differential expression of citrate metabolism genes. However, other researchers have reported that water stress leads to a decrease in pH in grape ‘Sauvignon blanc’ berries during fruit development (Gachons et al., 2005).

This article reviews the results of previous studies and provides a theoretical basis for fruit tree production and fruit quality regulation in water-deficient and arid areas. In addition, the changes in fruit sugar and organic acid contents, fruit sugar:acid ratio, associated metabolic enzyme activities, gene expression, and stress response mechanisms under water stress are summarized.

## Survey methodology

In this article, we used the Blyun database (https://www.blyun.com/), Web of Science (https://www.webofscience.com/wos/alldb/basic-search), Baidu Academic (https://xueshu.baidu.com/) and sci-hub (https://sci-hub.se/) to search for literature. The search keywords and their combinations included “soil water stress”; “water stress”; “water deficit”; “drought stress”; “irrigation effects”; “soil drought”; “abiotic stress”; “carbohydrates”; “sugar metabolism”; “glycolysis”; “sucrose metabolizing enzyme activities”; “sucrose metabolism”; “expression patterns of genes involved in sugar metabolism”; “TCA”; “organic acids”; “abscisic acid”; “ABA accumulation”; “sugars and acids”; “sucrose-synthase genes”; “organic acid degradation-related genes”. We collected and screened a large number of related studies based on their relevance to the topic, and excluded those unrelated. We aimed to describe the effects of water deficit on sugar and acid metabolism and the mechanism of response in fruits, so we also excluded articles with less relevance after identifying their focus by reading the abstract. It is important to note that this is a comprehensive but exhaustive literature review.

## Effect of water stress on sugar metabolism in fruit

### Sugar content in fruit

The content and composition of sugars in fruit are important indices of fruit quality, and the main prerequisites for synthesis of amino acids, pigments, and organic acids (Lester, Jifon & Makus, 2010; Zhang, Li & Cheng, 2010; Etienne et al., 2013). Glucose, fructose, and sucrose are the primary sugars accumulated in fruit. The sink strength of the fruit greatly affects the sugar content in the fruit. One index for estimation of sink strength is the activity of metabolic enzymes, which is strongly affected by the water content. Therefore, the water content of the plant is extremely important for sugar accumulation in fruit.

Many studies have found that reduced irrigation and water stress change the sugar content in fruit. For example, Rahmati et al. (2015) applied three severities of water stress—low-intensity stress (LS; 30% less water supply at harvest than cumulative crop transpiration), moderate stress (MS; 53%), and severe stress (SS; 64%)—from the mid-pit hardening stage (12 June) to the harvest stage (23 September) of 8-year-old peach (*Prunus persica* (L.) Batsch) ‘Alberta’ trees. In the LS treatment, the glucose and fructose contents decreased during fruit development, whereas no significant difference in glucose and fructose contents was observed among the treatments approaching fruit maturity. The SS treatment increased the concentrations of glucose, fructose, and sorbitol in the fruit flesh by approximately 12–70%, whereas the sucrose concentration was not affected by the water-stress treatment. During the harvest period, the fruit volume under the MS and SS treatments was 66% and 44% of that under the LS treatment. The fruit dry weight under the MS and SS treatments was 85% and 73% of that under the LS treatment. Although the fruit volume was significantly reduced under the MS and SS treatments, the dry weight and fructose, glucose, and sorbitol contents increased to a greater proportion. These results suggest that greater amounts of sugars accumulate in fruit under moderate and severe drought.

Wang et al. (2019) treated apple (*Malus × domestica* Borkh.) ‘Gala’ at stage I (from the young fruit stage to the mature stage) and stage II (from the fruit expanding stage to the mature stage B) with either LS (60–70% of field capacity [θ_f_] (Yang et al., 2015b)) or MS (50–60% θ_f_) treatments. In stage I, compared with the control (CK; 70–80% θ_f_), fructose, glucose, and sorbitol contents under the MS and LS treatments increased by 10% and 15%, 13% and 16%, and 56% and 107%, respectively. The sucrose content under the LS treatment increased by 27% in the harvesting period. During this stage, the enhanced activities of SuSy, sucrose phosphate synthase (SPS), and acid invertase (AI) under MS and the enhanced activities of SuSy and SPS under LS promoted the conversion of sucrose to fructose and glucose. In stage II, under the MS and LS treatments, the sugar contents in the fruit were increased compared with those of the CK in the harvesting period, especially fructose (26% and 12%), glucose (64% and 24%), and sorbitol (61% and 19%), whereas the sucrose content decreased by 23% and 17%, respectively. At this stage, the MS and LS treatments increased the activities of sorbitol oxidase (SOX) and AI in the fruit. Furthermore, LS reduced the fruit size in stage I but not in stage II. Thus, MS at stage I, and MS and LS at stage II, significantly increase the contents of fructose, glucose, and sorbitol in apple fruit, and the fruit size is reduced in stage I compared with that in stage II under exposure to water stress.

Alcobendas et al. (2013) observed that, compared with those under full irrigation, the contents of glucose and sorbitol in plants treated with deficient irrigation were higher in the mid- to late-maturing peach ‘Catherine’. However, no distinct differences in fructose and sucrose contents were detected. Kobashi, Gemma & Iwahori (2000) reported that sorbitol, sucrose, and total sugar contents in peach fruit increase under moderate water stress, whereas no significant difference is observed under severe water stress. Other studies have shown that water stress increases fruit sugar content (Seymen et al., 2021). Snchez-Rodrguez et al. (2012) examined the effect of grafting on fruit yield and quality under water stress in tomato (*Solanum lycopersicum* L.). The authors observed that a high content of sugars accumulated in fruit under moderate water stress when the drought-resistant ‘Zarina’ formed the rootstock and the drought-sensitive ‘Josefina’ was the scion.

In sum, the most strongly significant effect of water stress on fruit yield is on the average fruit weight rather than on the fruit number per plant, and the amount of sugars accumulated varies with the degree of water stress. The reason for these inconsistent results may be that moderate drought can lead to greater accumulation of sugars compared with that under mild and severe drought (Atkinson et al., 1998).

### Activity of enzymes associated with sugar metabolism

**Figure 1:**
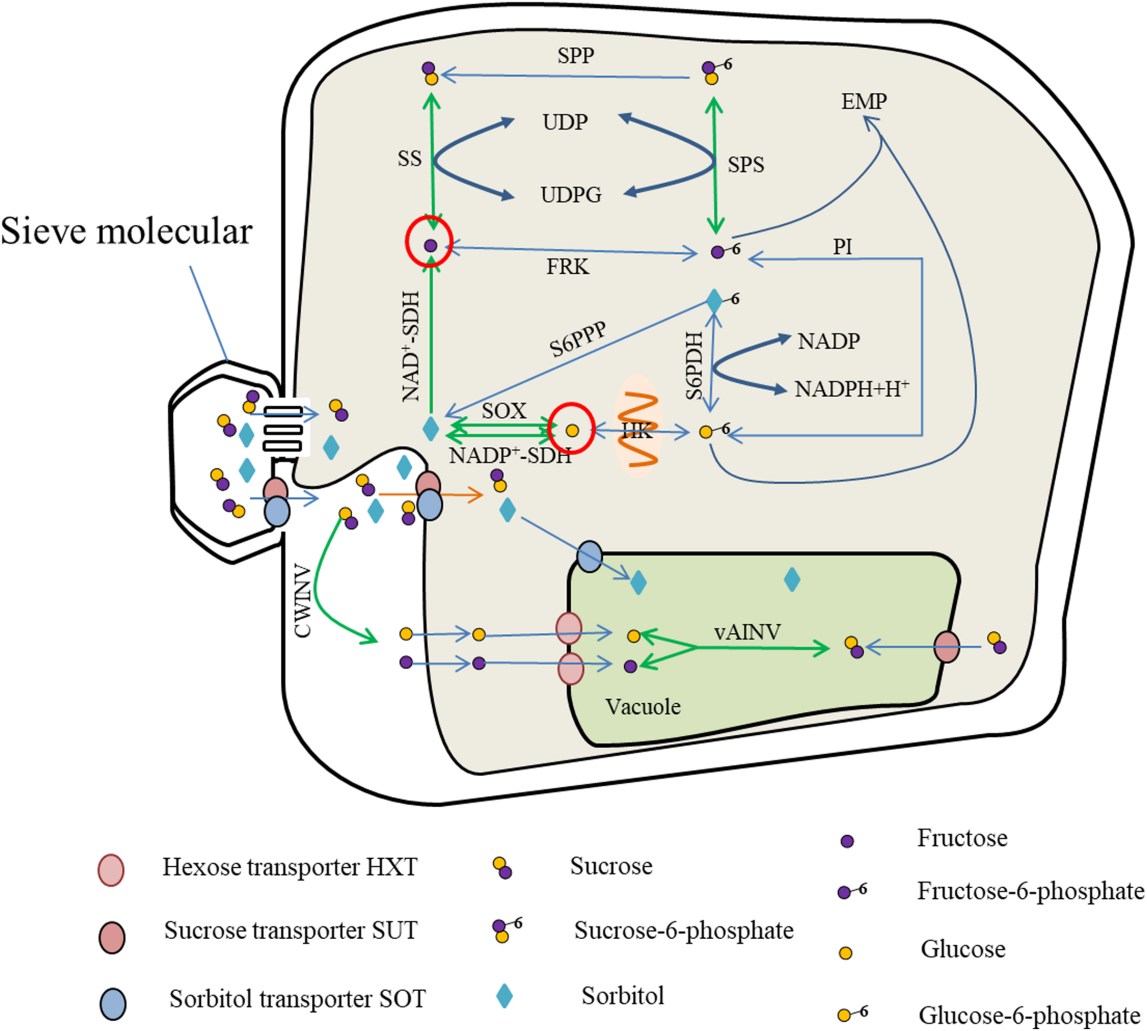
Sugar metabolism in fruit cell under water stress. The red circles indicate elevated fructose and glucose content (Rahmati et al., 2015; Alcobendas et al., 2013; Wang et al., 2019), while the green arrows represent enhanced activity of SS, SPS, vAINV, NAD^+^-SDH and SOX under water stress (Hockema & Etxeberria, 2001; Lu, Li & Jiang, 2009; Li et al., 2019; Wang et al., 2019). With enhanced SS activity, the rate of sucrose-fructose interconversion was accelerated (Hockema & Etxeberria, 2001), but SPP catalyzed irreversible reactions leading to sucrose-to-fructose conversion (Huber & Huber, 1996); with enhanced NAD^+^-SDH activity, the rate of sorbitol conversion to fructose was accelerated (Li & Li, 2005); with enhanced SPS activity, the rate of Sucrose-6-phosphate synthesis and decomposition was accelerated (Yang et al., 2002; Cornic, Ghashghaie & Fresneau, 2007), and due to enhanced SS activity, the rate of Sucrose-6-phosphate to sucrose, resulting in SPS-catalyzed Sucrose-6-phosphate synthesis, and the above reasons led to the increase of fructose content. The sorbitol transported into the fruit, due to the enhanced activity of SOX and NADP^+^-SDH (Li & Li, 2005; Wang et al., 2019), the conversion of sorbitol to glucose and the increase of glucose content.

Photosynthates are transported from the leaves to the fruit in the form of sucrose or sorbitol, and entry into the cells is mediated by protein carriers for carbohydrate metabolism. Metabolites such as fructose and glucose are either stored in the vacuoles *via* the hexose transporter (HXT) or catalyzed by soluble AI to produce sucrose. The sucrose can also contribute to the Embden–Meyerhoff–Parnas (EMP) glycolytic pathway for energy metabolism (Fig. 1).

The enzymes primarily involved in sucrose metabolism in fruit are SuSy, SPS, and INV (Li, Feng & Cheng, 2012). Of these enzymes, INV catalyzes the irreversible breakdown of sucrose to glucose and fructose. Depending on the pH for optimal activity, INV comprises neutral invertase (NINV) and AI, and AI is further dividable into soluble AI and insoluble AI. Soluble AI is localized in the cytoplasm or apoplastic space within the cell, whereas insoluble AI is bound to the cell wall. Sucrose synthase, comprising soluble SS in the cytoplasm and insoluble SS in the cell membrane, is mostly localized in the cytoplasm and catalyzes reversible reactions converting sucrose to uridine diphosphate glucose (UDPG) and fructose (Tanase et al., 2002; Yang et al., 2019). Sucrose phosphate synthase in the cytoplasm catalyzes UDPG and fructose 6-phosphate to produce sucrose phosphate, which is then irreversibly converted to sucrose under the catalysis of sucrose-phosphate phosphatase (Huber & Huber, 1996).

The enzymes associated with hexose metabolism are hexokinases, comprising glucokinase in the mitochondrial membrane and fructokinase in the cytoplasm. Glucokinase catalyzes the reversible phosphorylation of glucose, whereas fructokinase catalyzes the reversible phosphorylation of fructose (Petreikov et al., 2001; Renz, 1993).

Sorbitol is synthesized mainly in members of the Rosaceae. The enzymes that participate in sorbitol metabolism consist of sorbitol 6-phosphate dehydrogenase, SOX, and sorbitol dehydrogenase (SDH). Sorbitol 6-phosphate dehydrogenase catalyzes the conversion of glucose-6-phosphate and sorbitol-6-phosphate, and the activity of sorbitol-6-phosphate phosphatase converts sorbitol-6-phosphate into sorbitol. The decomposition of sorbitol into glucose is catalyzed by SOX. Sorbitol dehydrogenases comprise nicotinamide adenine dinucleotide phosphate-dependent sorbitol dehydrogenase (NADP+-SDH) and nicotinamide adenine dinucleotide-dependent sorbitol dehydrogenase (NAD+-SDH). Of these enzymes, NAD+-SDH catalyzes the decomposition of sorbitol to form fructose, and NADP+-SDH and SOX primarily catalyze the conversion of sorbitol to glucose (Yamaguchi, Kanayama & Yamaki, 1994).

Under water stress, SuSy activity in the fruit increases, thus accelerating the rate of sucrose metabolism (Hockema & Etxeberria, 2001). Under light water stress, the activities of SuSy and AI increase, but under moderate water stress only the activity of SuSy increases. In addition, light and moderate water stress boost the activity of SOX, and thereby promote conversion of sorbitol into glucose in apple (Wang et al., 2019). The activities of SuSy and INV in tomato fruit increase under long-term water stress (Lu, Li & Jiang, 2009; Li et al., 2019). Increased SDH activity under drought accelerates the rate of sorbitol decomposition (Li & Li, 2005). The activity of SPS increases in rice (*Oryza sativa* L.) and durum wheat (*Triticum durum* L.) under water stress *(Yang et al., 2002*; *Cornic, Ghashghaie & Fresneau, 2007)*. As the activities of SuSy and sucrose INV (especially sucrose AI) increase in the leaves of coffee (*Coffea canephora* Pierre ex A.Froehner var. *kouilouensis* De Wild.), the content of hexose is also increased (Praxedes et al., 2007). Therefore, the activities of SuSy, SOX, SDH, INV, and SPS are generally increased under water deficit. This may be the cause of hexose accumulation.

The ectopic expression of apple *MdSUT2* in tomato improves the soluble sugar content in the transformed lines and enhances tolerance to adverse environmental factors, such as soil salinization and drought (Ma et al., 2016). In addition, drought stress induces expression of the monosaccharide transporter *TMT1* on the vacuolar membrane of *Arabidopsis thaliana* (Wingenter et al., 2010). The genes *TaSUT1A*, *TaSUT1B*, and *TaSUT1D* are highly expressed in the glume, stem, grain, and seeds during the grain filling stage. Such expression promotes sugar absorption and source–sink transport (Aoki et al., 2004; Aoki et al., 2006). Under mild water deficiency, an increase in glucose content is associated with significant decrease in expression of the *VvHT1* hexose transporter gene. Under severe water stress, the expression levels of the monosaccharide transporter *VvHT5*, sucrose carrier *VvSUC11*, vacuolar invertase *VvGIN2*, and *ASR* (abscisic acid [ABA], stress, and ripening-induced) genes increase in grape (Medici, Laloi & Atanassova, 2014).

### Expression of genes associated with sugar metabolism

Sugar accumulation in fruit is associated with the expression of enzymes involved in sucrose metabolism. Islam et al. (2014) observed that, compared with those of the control, under moderate water stress the transcript levels of *CitSUS1* and *CitSUS3–5* (no obvious phenotypic change was observed in the leaves and other tissues) decrease significantly and those of *CitSUS6* are slightly reduced, whereas the *CitSUS2* transcript level increases almost 2.5 times in the fruit segment membrane of *Citrus unshiu* (Swingle) Marcow. In fruit juice sacs, *CitSUS2*, *CitSUS4*, and *CitSUS6* transcript levels show a trend to increase compared with those of the control. The levels of *CitSUS2* transcripts increase 2-fold and *CitSUS4* transcripts increase 3.6-fold, which is significantly higher than those in the control. The *SS* genes are the main genes involved in plant sucrose metabolism. In most dicotyledonous plants, there are two non-allelic genes, *SS1* and *SS2*. Osmotic stress induces expression of SuSy in the resurrection plant (*Craterostigma plantagineum* Hochst.), non-dormant wheat (*Triticum aestivum* L. ‘Trémie’), and tomato ‘Liaoyuan Duoli’ (Kleines et al., 1999; Niedzwiedz-Siegien et al., 2004; Lu, Li & Jiang, 2010). Interestingly, Baud, Vaultier & Rochat (2004) reported that *AtSS3* was expressed in various organs of *Arabidopsis* after leaf dehydration. Invertase-related genes display different expression patterns under water stress and the expression of *INV* genes differs under various degrees of water stress. A similar finding has been reported for tomato, in that the expression levels of *AI* and mRNAs increase under water stress (Lu, Li & Jiang, 2009; Li et al., 2019). Trouverie et al. (2004) showed that the expression level of *INV2* in the vacuole of maize (*Zea mays* L.) leaves is elevated under moderate water stress and thus AI activity is increased in mature maize leaves. Sharifi et al. (2018) observed that the expression levels of *SPS* and *INV* genes increases under drought stress. However, Mclaughlin & Boyer (2004) reported that, under drought stress, the expression levels of cell wall INV, vacuolar INV, and SuSy decrease in the ovary of maize. These differences may result from the differential expression of INV2 (Pelleschi et al., 1999).

## Effect of water stress on organic acid metabolism in fruit

### Content of organic acids in fruit

Both the acid content of the fruit and the sugar:acid ratio are important indicators of fruit quality. The principal site of organic acid synthesis is the mitochondria and the main synthetic pathway is the TCA cycle (Etienne et al., 2013). In the process of fruit ripening, organic acids are gradually metabolized and utilized through the TCA pathway, glycolytic pathway, and gluconeogenesis. The diverse species share the same TCA pathway. Based on the predominant constituent organic acid, fruit can be roughly divided into tartaric acid, malate, and citrate types.

In grape berries, organic acid synthesis and accumulation are initiated in the stages of pollination and fruit set, and the main location for their metabolism is the mitochondria. Ussahatanonta, Jackson & Rowe (1996) studied the effects of nutrient and water stress on vegetative and reproductive growth in grape ‘Cabernet Sauvignon’. These authors reported that under intermittent water stress (watering three times per week), the number and total weight of berries decreased by 21.9% and 12.9%, respectively. Compared with the results from the intermittent water stress treatment, the contents of malic acid and tartaric acid under treatment with water and nutrient sufficiency (applying a standard concentration of fertilizer) increased by 23.9% and 16.6%, respectively. Cholet et al. (2016) conducted a comparative study of the tartaric acid pathway in grape ‘Ugni blanc’ berries under two vintages with contrasting climatic conditions (one hotter and drier, the other colder and wetter). Under the hotter and drier climate, the tartaric acid content of fruit in the harvest period was considerably higher than that under the cooler and wetter climate, which indicated that a dry climate is more favorable for tartaric acid accumulation. Aguado et al. (2012) observed that, compared with the control (irrigation with 100% of crop evapotranspiration), the weight of orange ‘Navelina’ fruit and juice under water deficit (irrigation with 75% of the control) increased by 17% and 21%, respectively, but no significant change in titratable acidity was detected. Qi et al. (2003) reported that water deficit increases the organic acid content of tomato fruit. In addition, compared with LS and MS treatments, SS treatment significantly increases citric acid and quinic acid concentrations throughout fruit development; with intensification of drought, the total organic acid and total non-structural carbohydrate concentrations increase (Rahmati et al., 2015). Overall, the organic acid content of fruit increases under water stress.

### Activity of enzymes associated with organic acid metabolism

**Figure 2:**
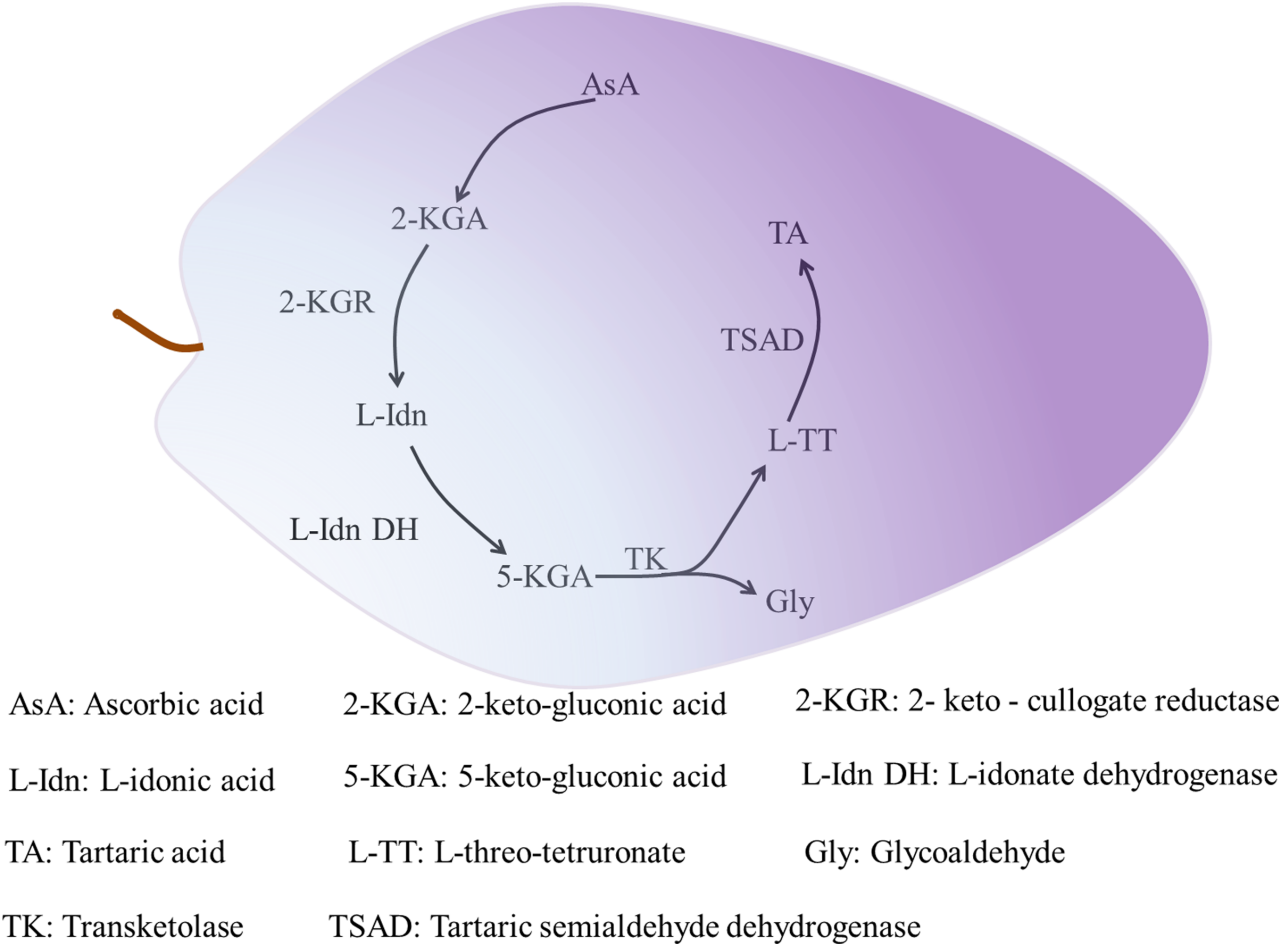
The synthesis of tartaric acid in grape. The initial substrate of tartaric acid synthesis is ascorbic acid, and the synthesis reaction takes place in the fragment of ascorbic acid C4-C5, and tartaric acid in grapes is synthesized through the intermediate metabolic pathways (Jia et al., 2019). The related enzymes mainly include 2-KGR, L-IDN DH, TK and TSAD, among which L-IDN DH is the rate-limiting enzyme. The process of 2-KGA synthesis by AsA is not clear. 2-KGA is reduced to L-Idn by 2-KGR (Jia et al., 2019), then L-IDO is oxidized to 5-KGA by L-Idn DH (Debolt, 2006). TK catalyzes the cleavage of 5-KGA between C4 and C5 to produce L-TT and Gly. Finally, L-TT generates TA by the catalysis of TSAD (Debolt, 2006).

Organic acids accumulate gradually during plant growth. Two types of proton pumps at the tonoplast, vacuolar-type ATPase and vacuolar pyrophosphatase, drive entry of the synthesized organic acids into the vacuole for storage by means of transporters and specialized cation channels (Ratajczak, 2000; Meyer et al., 2011). Tartaric acid only accumulates in the fruit of grape and *Pelargonium* species (Stafford, 1959). Tartaric acid is synthesized in leaves and immature fruit of grape, and in the leaves and pods of *Pelargonium* plants, but not in mature fruit. In grape, tartaric acid is mainly synthesized as the result of cleavage of the C4–C5 group of AsA and involves the metabolic enzymes 2-keto-gulonate reductase (2-KGR) (Jia et al., 2019), L-IDN DH (Debolt, Cook & Ford, 2006), transketolase, and tartaric semialdehyde dehydrogenase (TSAD) (Debolt, 2006). Tartaric acid synthesis progresses as follows. Ascorbic acid is hydrolyzed and oxidized to produce 2-keto-gluconic acid (2-KGA). Then, 2-KGR catalyzes 2-KGA to produce L-idonic acid (L-IDN), and the rate-limiting enzyme L-IDN DH oxidizes L-IDN to form 5-keto-gluconic acid (5-KGA). Transketolase then catalyzes 5-KGA to produce L-threo-tetruronate (L-TT) and glycoaldehyde, and finally TSAD catalyzes L-TT to produce tartaric acid (Fig. 2) (Debolt, Cook & Ford, 2006). Given its restricted distribution among flowering plants, few studies have investigated tartaric acid metabolism. Other organic acids, except some malate synthesized in the cytoplasm, are synthesized in mitochondria through the TCA cycle (Etienne et al., 2013).

The enzymes that participate in malate metabolism in fruit are malate dehydrogenase (MDH), malic enzyme (ME), phosphoenolpyruvate carboxylase (PEPC), and phosphoenolpyruvate carboxykinase (PEPCK). Malate dehydrogenase catalyzes reversible reactions and is mainly localized in the cytoplasm (Cyt-MDH, the most important isozyme), mitochondria, and chloroplasts (Yao et al., 2011). Based on the characteristics of the coenzymes, there are two forms of ME: NAD-ME and NADP-ME. The form NAD-ME is mainly localized in mitochondria, whereas NADP-ME is predominantly in the cytoplasm and plastids.

**Figure 3:**
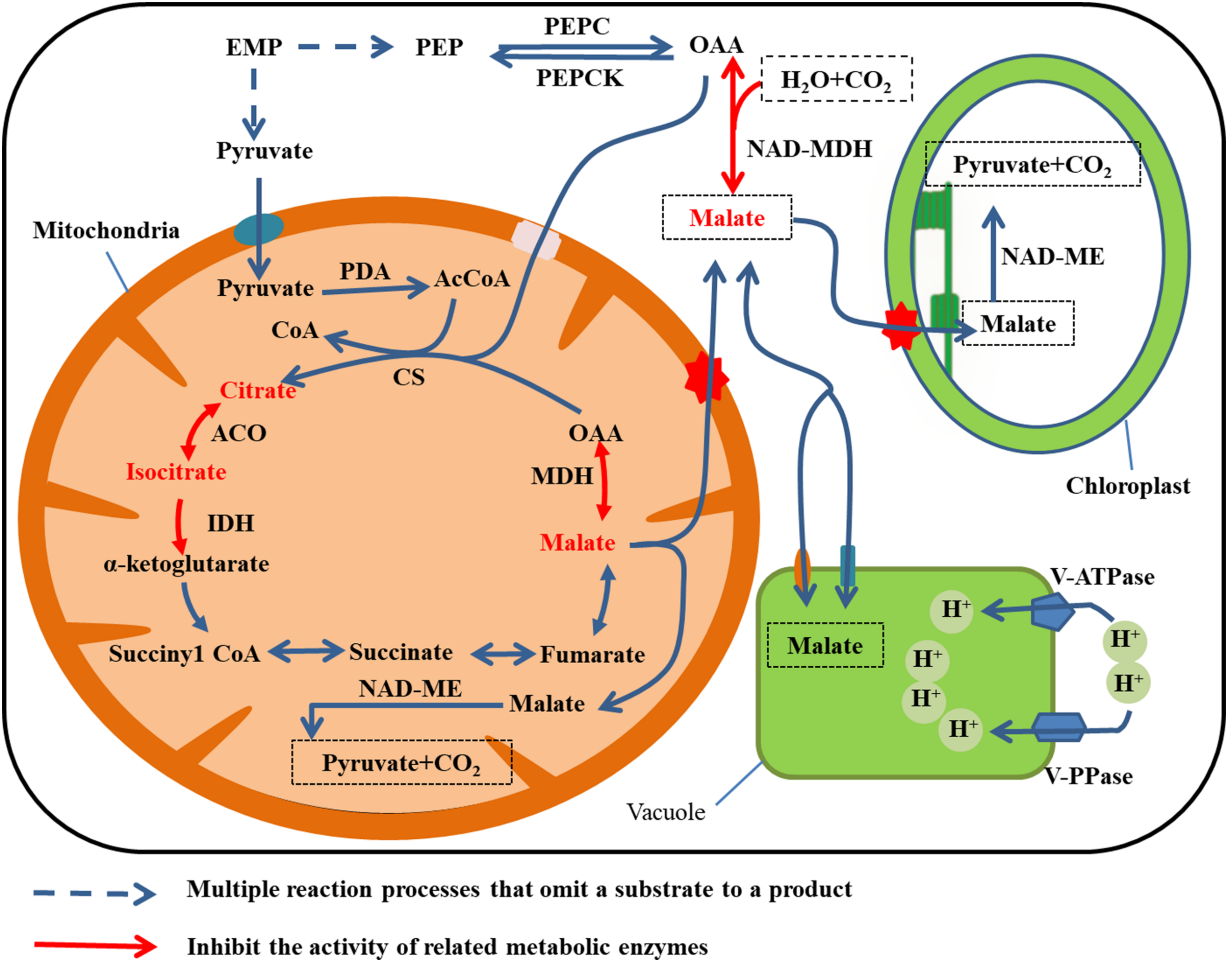
Malate metabolism in fruit cell under water stress. The red font: material accumulation (Jiang et al., 2014). Dark blue dashed arrows: omission of the multiple reaction process of the substrate formation product; red arrow: inhibition of related metabolic enzyme activities. Under water stress, ACO, IDH and MDH enzyme activities are inhibited and citrate, isocitrate and malate metabolism rates are reduced (Jiang et al., 2014), leading to accumulation. Malate was translocated to vacuole storage and its content increases.

The synthesis and degradation pathways for malic acid are shown in Fig. 3. Two pathways are involved in malate anabolism. In one pathway, the phosphoenolpyruvate (PEP) produced by EMP in the cytoplasm generates oxaloacetic acid (OAA) under the activity of PEPC, and then NAD-malate dehydrogenase (NAD-MDH) catalyzes OAA to produce malate. In the second pathway, malate is synthesized through TCA in the mitochondria (Etienne et al., 2013). Malate is degraded by two mechanisms. Cytoplasmic NADP-ME catalyzes malate to produce OAA, which is then irreversibly converted to PEP under the catalysis of PEPCK (Bahrami et al., 2001). Alternatively, NAD-ME catalyzes degradation of malate to pyruvate and CO_2_ in the mitochondria and chloroplasts. The latter reaction is the main means of malic acid degradation (Sweetman et al., 2009).

The primary enzymes involved in citrate synthesis are citrate synthase (CS) in the mitochondria (O’Hara, Slabas & Fawcett, 2002; Lin et al., 2016), and aconitase (ACO) in the cytoplasm and mitochondria. Aconitase reversibly catalyzes the conversion of citrate and isocitrate. The principal enzymes for citrate degradation are isocitrate dehydrogenase (IDH), glutamate dehydrogenase (GAD), glutamine synthetase (GS), and ACO (Tadeo et al., 2008; Chen et al., 2013; Liao et al., 2019). Isocitrate dehydrogenase can be divided into NAD-IDH (localized in mitochondria) and NADP-IDH (localized in the cytoplasm).

**Figure 4:**
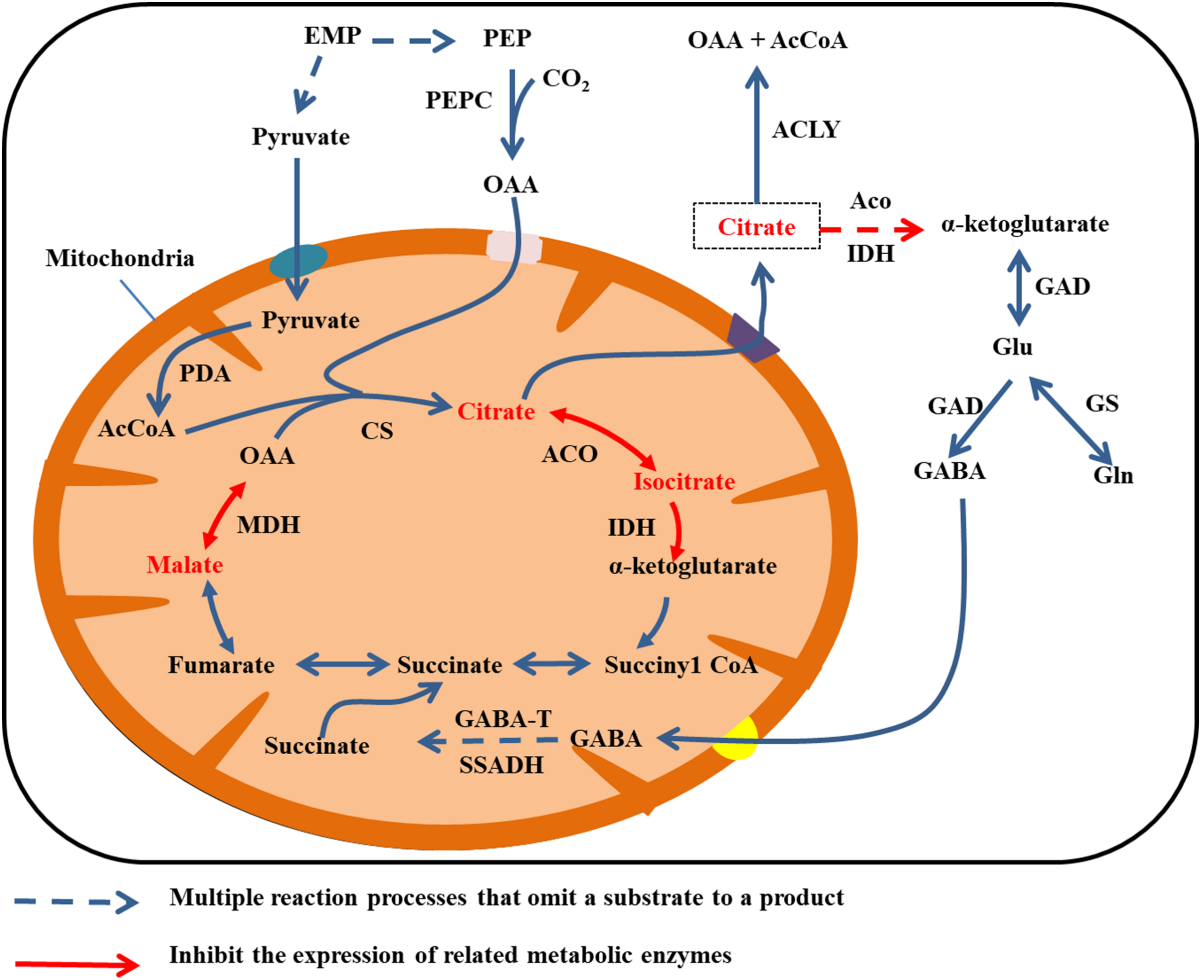
Cirrate metabolism in fruit cell under water stress. The red font: material accumulation (Jiang et al., 2014). Dark blue dashed arrows: omission of the multiple reaction process of the substrate formation product; red arrow: inhibition of related metabolic enzyme activities; red dashed arrows: multiple reaction processes in which the substrate forms a product are omitted and the catalyticase activity of at least one step of the reaction is inhibited. Under water stress, ACO, IDH and MDH enzyme activities are inhibited and citrate, isocitrate and malate metabolism rates are reduced (Jiang et al., 2014), leading to accumulation. Citrate was transported to the cytoplasm for metabolism.

Citrate is mainly degraded in the cytoplasm by two mechanisms. Aconitase and IDH catalyze citric acid to produce α-ketoglutarate, which gives rise to glutamate (Glu) under the activity of GAD. In turn, GAD catalyzes glutamate to produce GABA, and GABA enters the mitochondria. Then GABA transaminase (GABA-T) and succinate semialdehyde dehydrogenase (SSADH) catalyze GABA to produce succinate, and finally succinate enters the TCA cycle (Cercós et al., 2006; Michaeli et al., 2011). The second type of degradation involves the breakdown of citrate to OAA and acetyl coenzyme A by the activity of ATP citrate lyase (Giovannoni, 2004). In addition, citrate can be degraded *via* the glutamine pathway. By this mechanism, glutamine synthetase (GS) catalyzes glutamate to produce glutamine (Fig. 4) (Cercós et al., 2006).

The activity of MDH decreases under water stress. Malic acid and citric acid accumulate in juice cells of ripe citrus fruit after mulching, and the organic acid content in fruit flesh increases with continuing water deficiency after mulching. Water shortage from surface mulching may reduce the activity of NADP-IDH and cytoplasmic ACO, resulting in the slow degradation of citric acid. Jiang et al. (2014) observed that mulching film had little effect on the activities of PEPC and CS, but decreases the activities of cytoplasmic ACO and cytoplasmic IDH during fruit development and at fruit maturity.

### Expression of genes associated with organic acid metabolism

Previous studies have focused on malic acid metabolism-related gene expression at different stages. Few studies have investigated the effect of water stress on the expression of tartaric acid and malic acid metabolism-related genes, whereas studies on the expression of citric acid metabolism-related genes in citrus fruit under water stress has been studied in more detail.

The *Citrus* genome contains two CS genes, *CitCS1*, and *CitCS2*. According to the relevant studies, the increase in citric acid content in fruit of Wenzhou mandarin (*Citrus reticulata* Blanco ‘Unshiu’) under 40% water stress may be due to increased expression of *CitCS*. In addition, expression of *CitIDH* and *CitIDHI* in Wenzhou mandarin fruit increases during late drought, but in other periods the expression levels decrease to different degrees; overall, *CitIDH* and *CitIDHI* mainly increase compared with the control. However, *CitNADPIDH* rapidly increases in late drought but decreases in other periods of drought. Accumulation of citric acid is associated with ACO activity. For example, Liu et al. (2007) observed that decrease in *ACO* expression led to accumulation of citrate in late-ripening navel orange fruit, and Morgan, Osorio & Gehl (2013) showed that inhibition of the expression of *SlAco3a* and *SlAco3b* in tomato fruit decreases ACO activity and increases the citrate content.

## Signal transduction of water stress and sugar and acid metabolism in fruit

Under water stress, plant cells perceive and receive stress signals through signal sensors, and convert the extracellular signals into intracellular signals for transduction. By this process, the second messenger, produced in plants with the stimulation of water stress, plays an important role in signal transduction.

**Figure 5:**
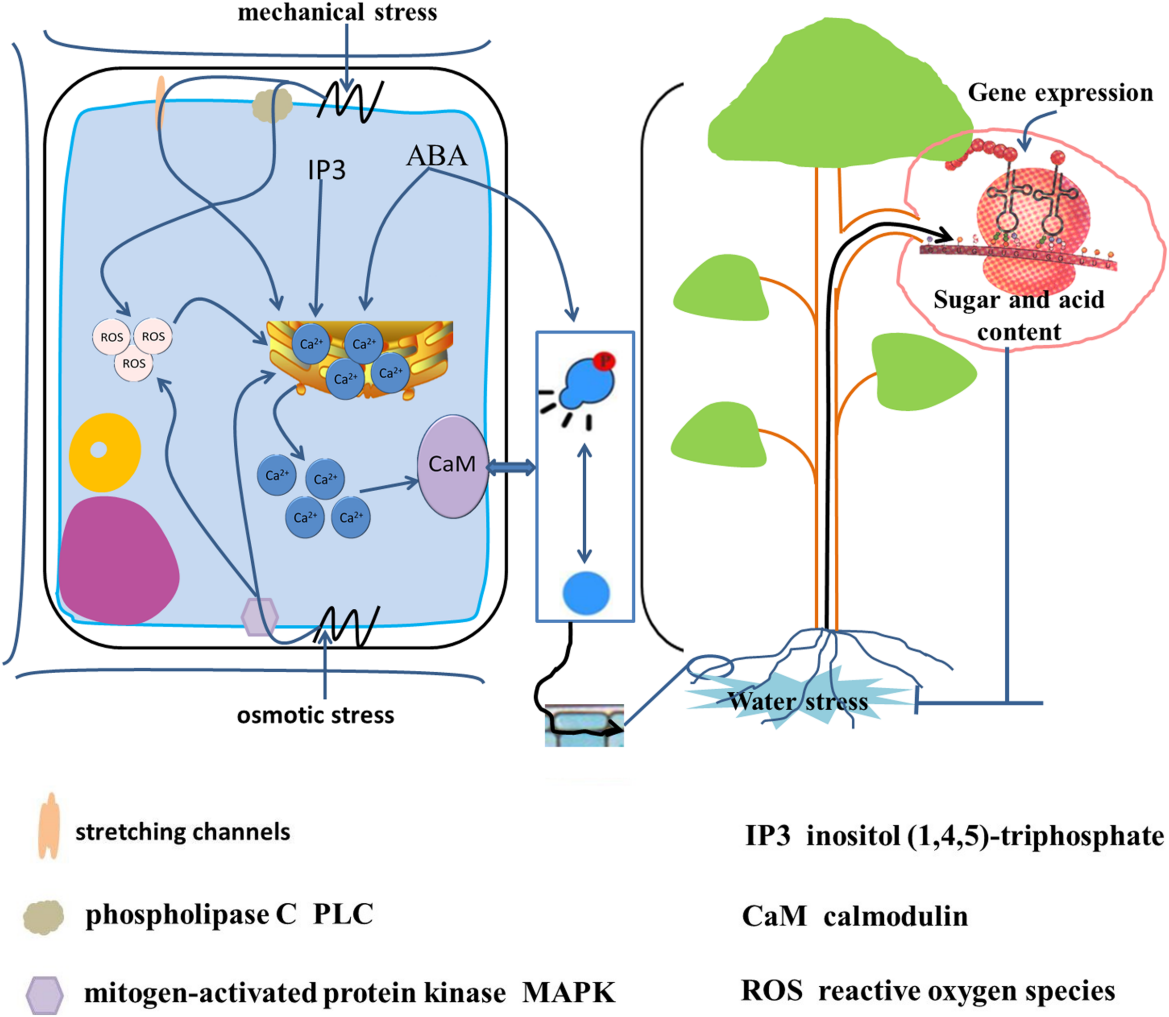
Signal transduction from root to fruit and water stress response in the root cell. Water stress is translated into osmotic stress, mechanical stress and oxidative stress (Gong et al., 2020; Niedzwiedz-Siegien et al., 2004; Cao et al., 2015). Mechanical stress triggered by cellular water loss is recognized by phospholipase C (PLC) and stretch channels and on the plasma membrane, and osmotic stress is recognized by mitogen-activated protein kinase (MAPK), leading to an increase in intracellular free Ca^2+^ concentration and the production of reactive oxygen species (ROS) (Gong et al., 2020; Dastogeer et al., 2017). In addition, water stress increases intracellular inositol (1,4,5)-trisphosphate (IP3) and ABA synthesis, which induces endoplasmic reticulum Ca^2+^ entry into the cytoplasm. High intracellular concentrations of ABA and Ca^2+^-receiving calmodulin caused reversible phosphorylation of proteins (Iwata et al., 1998), induced gene expression, and caused changes in sugar and acids metabolism.

Upon exposure of a plant to water stress, the initial stress signals are translated into osmotic stress signals, mechanical stress signals, and oxidative stress signals (Fig. 5) (Niedzwiedz-Siegien et al., 2004; Cao et al., 2015; Gong et al., 2020). The water deficit in cells leads to a change in turgor pressure and results in mechanical pressure. Phospholipase C and mechanosensitive channels recognize the mechanical pressure on the plasma membrane, and stimulate a brief increase in Ca^2+^ concentration within the cell (Gong et al., 2020). The change in turgor pressure also leads to recognition of osmotic stress by mitogen-activated protein kinase (MAPK). Both osmotic stress and mechanical stress lead to increase in the intracellular free Ca^2+^ concentration and the production of reactive oxygen species (Dastogeer et al., 2017; Gong et al., 2020). In addition, water stress increases the activity of phospholipase C and promotes the synthesis of inositol (1,4,5)-triphosphate (IP3). The IP3 molecule is also a second messenger and induces transport of endoplasmic reticulum Ca^2+^ into the cytoplasm to increase the intracellular Ca^2+^ concentration. Calmodulin receives high cytoplasmic Ca^2+^ concentrations leading to reversible phosphorylation of proteins (Iwata et al., 1998). Protein phosphorylation promotes SPS enzyme activity in banana (*Musa*) (Hubbard, Pharr & Huber, 1990). The drought-induced protein kinase MdCIPK22 interacts with and phosphorylates Md-SUT2.2, thus enhancing MdSUT2.2 protein stability and promoting accumulation of soluble sugars in the vacuole. Ultimately, stress resistance and fruit quality are enhanced *(Ma et al., 2019)*.

Water stress induces gene expression in the ABA biosynthesis pathway, which is an important signal transduction pathway (Jia et al., 2002). It is generally considered that ABA can be synthesized in all plant tissues (Gong et al., 2020), but under water stress ABA is only synthesized in root cells owing to changes in plant metabolism. Under water stress, a large amount of ABA, an intracellular messenger, is synthesized in root cells (Christmann et al., 2005), which is sensed by ABA-binding sites (Gong et al., 2020). As a consequence, protein kinase gene expression is induced, the activity of calcium-dependent kinase is increased, and protein phosphorylation is regulated (Li et al., 2019). In addition, ABA stimulates elevation in the concentration of the second messenger Ca^2+^. Soluble sugars accumulate under drought (Falahi et al., 2018). In almost all studied grape cultivars, increase in ABA content promotes sugar accumulation in the fruit under water stress (Castellarin et al., 2007). Similarly, the concentration of fructose and glucose in fruit is significantly increased in response to spray application of exogenous ABA onto tomato leaves (Barickman, Kopsell & Sams, 2017). Sugars act as osmotic regulators in plants and their accumulation is also a result of genetic induction (Izanloo et al., 2008; Ambavaram et al., 2014; Yang et al., 2015a). Giribaldi et al. (2010) observed that ABA affected the activity of a vacuoles-inverting enzyme (GIN1) and ME during fruit ripening. Furthermore, ABA regulates the expression of sugar-responsive genes through the downstream signaling element ABI4. In addition, ABA regulates sugar metabolism by inducing the transcription of relevant genes by signaling in the nucleus (Rook et al., 2006).

## Conclusion

Studies on the effect of water stress have shown that sugar and acid contents in different fruit vary under different intensities of water stress. Monitoring the activities of catalytic enzymes indicates that water stress changes the activity of enzymes involved in sugar and acid metabolism in fruit, and thus change the rate of sugar and acid metabolism. Advanced technologies have been utilized to study the reasons for the changes in sugar and acid contents in fruit at the molecular level, and indicate that water stress also stimulates changes in the expression level of genes associated with sugar and acid metabolism. In addition, many studies on water stress signal perception, signal transduction, and differential expression of stimulus-related genes have revealed the effects of water stress on plant physiological metabolism.

Although substantial progress has been achieved, some limitations remain. First, previous studies on the effect of water stress on fruit sugar content have mostly focused on the changes in sucrose, glucose, and fructose contents and related metabolic enzymes and genes, whereas few studies have investigated other types of sugars. Second, studies of the effect of water stress on fruit acid content have predominantly concentrated on malate and citrate, and investigations of tartaric acid have focused on its synthesis pathways. Lastly, although it has been concluded that the changes in sugar and acid contents in different fruit vary according to the degree of water stress, the molecular mechanism responsible is unclear.

Considerable research is still required to elucidate the effects of water stress on fruit sugar and acid metabolism. We consider that, with continuing research effort, a more complete picture of the mechanism by which water stress influences fruit sugar and acid metabolism will be assembled in the near future.

## Additional Information and Declarations

### Data Availability

The following information was supplied regarding data availability: This is a literature review.
